# RNA-Based Anti-Inflammatory Effects of Membrane Vesicles Derived from *Lactiplantibacillus plantarum*

**DOI:** 10.3390/foods13060967

**Published:** 2024-03-21

**Authors:** Shino Yamasaki-Yashiki, Fumie Kawashima, Azusa Saika, Ryota Hosomi, Jun Kunisawa, Yoshio Katakura

**Affiliations:** 1 Department of Life Science and Biotechnology, Faculty of Chemistry, Materials and Bioengineering, Kansai University, 3-3-35 Yamate-cho, Suita, Osaka 564-8680, Japan; fumiekawashima23@gmail.com (F.K.); hryotan@kansai-u.ac.jp (R.H.); katakura@kansai-u.ac.jp (Y.K.); 2 Laboratory of Vaccine Materials, Laboratory of Gut Environmental System, Microbial Research Center for Health and Medicine, National Institutes of Biomedical Innovation, Health and Nutrition (NIBIOHN), Ibaraki, Osaka 567-0085, Japan; azusa_saika@imcb.a-star.edu.sg (A.S.); kunisawa@nibiohn.go.jp (J.K.); 3 Institute of Molecular and Cell Biology, Agency for Science, Technology and Research, Singapore 138667, Singapore

**Keywords:** membrane vesicle, lactic acid bacteria, RNA, anti-inflammatory effect, gut

## Abstract

Bacteria generally release extracellular membrane vesicles (MVs), which are nanoparticles that play important roles in bacterial–bacterial and bacterial–host communication. As probiotics, lactic acid bacteria provide diverse health benefits to their hosts. In this study, we found that the Gram-positive lactic acid bacteria *Lactiplantibacillus plantarum* subsp. *plantarum* NBRC 15891 produce high amounts of MVs (*Lp*MVs), and that *Lp*MVs inhibit interleukin (IL)-8 production induced by lipopolysaccharide in intestinal epithelial HT29 cells. Heat- or UV-killed bacterial cells did not exhibit anti-inflammatory effects, and there was no uptake of these bacterial cells; contrarily, *Lp*MVs were taken up into the cytoplasm of HT29 cells. Small RNAs extracted from *Lp*MVs also suppressed IL-8 production in HT29 cells, suggesting that RNAs in the cytoplasm of bacterial cells are encapsulated in the MVs and released from the cells, which may be delivered to HT29 cells to exert their anti-inflammatory effects. In addition, administration of *Lp*MVs to mice with dextran sodium sulfate-induced colitis alleviated colitis-induced weight loss and colon length shortening, indicating that *Lp*MV intake is likely to be effective in preventing or ameliorating colitis.

## 1. Introduction

Inflammatory bowel disease (IBD) is a general term used to describe diseases that cause intestinal inflammation, including Crohn’s disease and ulcerative colitis. The causes of IBD are diverse and include environmental and genetic factors, such as disruption of the gut microbiota or intestinal barrier and immunodeficiency [1,2]. Currently, the mainstay treatments include 4-aminosalicylic acid and steroids, which suppress inflammatory cytokine production; however, there is no fundamental cure [3]. In the intestinal tract of patients with IBD, inflammation is triggered by excessive immune cell responses due to loss of the mucin layer, disruption of tight junctions of epithelial cells, and invasion of intestinal bacteria into the mucosal lamina propria [2]. Inflammatory cytokines, including interleukin (IL)-8, are chemokines that activate leukocyte lineage cells, such as neutrophils and basophils, which are involved in neutrophil chemotaxis. The excessive release of inflammatory cytokines results in cytotoxicity, leading to severe IBD [4].

Probiotics such as lactic acid bacteria and bifidobacteria have been reported to be effective in preventing and alleviating IBD [5,6]. For example, the administration of *Bifidobacterium adolescentis* to mice with dextran sulfate sodium (DSS)-induced colitis improved the remodeling of the gut microbiota and resulted in colitis improvement [7]. In addition, the administration of *Bifidobacterium longum* subsp. *longum* YS108R improved DSS-induced colitis in mice by stimulating the expression of tight junction proteins and promoting mucin production [8]. In terms of direct effects on immune cells, lipoteichoic acid derived from *Lactobacillus reuteri* DMSZ 8533 suppressed inflammation induced in macrophage-like cells [9], while *Lactococcus lactis* ML2018 inhibited nitric oxide production and improved DSS-induced colitis in macrophage-like cells [10]. These findings highlight a significant interaction between probiotics and the host immune system.

On the other hand, the functionality of extracellular membrane vesicles (MVs) produced by bacteria has garnered significant attention in recent years. Generally, both Gram-negative and -positive bacteria produce MVs that range from 20 to 500 nm in diameter. These MVs contain proteins, nucleic acids, and cell wall components that are found in the parent cells [11,12]. MVs play a crucial role in the communication between bacterial cells, such as quorum sensing. They are also involved in delivering pathogenic factors to the host and regulating the host’s immune system [11,12]. Probiotics, including lactic acid bacteria and bifidobacteria, also produce MVs [13,14,15,16]. We have previously reported that MVs derived from *Latilactobacillus sakei* subsp. *sakei* NBRC 15893 [14,17] and *Limosilactobacillus antri* JCM 15950 [15] enhance the production of immunoglobulin A in a manner similar to that of the parent cells. Since MVs inherit the components of their parent cells, it is likely that probiotic MVs also exhibit anti-inflammatory activities and that the appropriate intake of probiotic MVs could be applied to the prevention and treatment of IBD. Moreover, MVs are considerably smaller than bacterial cells and have a completely different surface structure from that of Gram-positive bacterial cells, which are enveloped in peptidoglycan. Although MVs are expected to act on the host in different ways from bacterial cells, previous studies on probiotics in fermented foods and commensal intestinal bacteria have overlooked MV-mediated bioactivity.

In the present study, to apply MVs for the prevention and alleviation of IBD, probiotic-derived MVs with anti-inflammatory effects in vitro were screened from several lactic acid bacteria and bifidobacteria, and their effects were demonstrated using mice with DSS-induced colitis. We found anti-inflammatory activity in MVs derived from *Lactiplantibacillus* (previously *Lactobacillus*) *plantarum* subsp. *plantarum* NBRC 15891 (*Lp*MVs), based on the inhibition of IL-8 produced by lipopolysaccharide (LPS) derived from *Escherichia coli* in HT29 intestinal epithelial cells. Furthermore, the mechanism by which MVs, which can be considered natural liposomes, exert their anti-inflammatory effects on HT29 cells was investigated in terms of the uptake of MVs by HT29 cells and the components of *Lp*MVs. Finally, *Lp*MVs were administered to mice with DSS-induced colitis, and the anti-inflammatory effects of *Lp*MVs in the prevention and treatment of IBD were evaluated.

## 2. Materials and Methods

### 2.1. Bacterial Culture

*L. plantarum* subsp. *plantarum* NBRC 15891 and *Leuconostoc mesenteroides* subsp. *sake* NBRC 102481 were purchased from the Biological Resource Center, NITE (Tokyo, Japan). *L. antri* JCM 15950, *Lactobacillus gasseri* JCM 1131, *B. longum* subsp. *infantis* JCM 1222, *B. longum* subsp. *longum* JCM 1217, and *Bifidobacterium breve* JCM 1192 were purchased from the RIKEN BioResource Center (Ibaraki, Japan). *L. plantarum* NCIMB 8826 was purchased from the National Collection of Industrial, Food and Marine Bacteria (Aberdeen, UK). Lactic acid bacteria were anaerobically cultured in de Man, Rogosa, and Sharpe (MRS) medium using the culture-tech (As One, Osaka, Japan). Bifidobacteria were anaerobically cultured in MRS medium containing 0.05% cysteine, using the culture-tech system. The incubation temperatures are listed in Table S1. The cell concentration was determined by measuring the optical density at 660 nm (OD_660_) of the culture broth. The concentration of viable cells was measured using the colony counting method.

For the preparation of heat- or UV-killed cells, *L. plantarum* subsp. *plantarum* NBRC 15891 was cultured for 24 h. Heat-killed cells were prepared by heating bacteria at 80 °C for 10 min. UV-killed cells were prepared by UV-irradiating bacteria suspended in PBS at 1 mW/cm^2^ for 1 h using a UV lump SUV-16 (As One, Osaka, Japan). The heat- and UV-killed cells were dried under reduced pressure at room temperature (18–23 °C).

### 2.2. Preparation of MVs

Briefly, the cell supernatant was collected via centrifugation (9500× *g*, 25 °C, 10 min) and filtered (cellulose acetate; pore size 0.22 µm). Subsequently, the filtrate was ultracentrifuged (150,000× *g*, 4 °C, 2 h), and the precipitate was then suspended in 10 mM HEPES buffer containing 0.85% NaCl (HEPES-NaCl, pH 6.8) and used as the crude MV fraction. If purification was required, 200 µL of the crude MV fraction was applied to a Sephacryl S-500HR column (φ1.5 × 10 cm; Cytiva, Tokyo, Japan) and developed with HEPES-NaCl. Fractions containing MVs were collected, and HEPES-NaCl was replaced with saline to prepare MVs for animal experiments. MVs were observed using a transmission electron microscope (TEM) JEM-1400 (JEOL, Tokyo, Japan), as described previously [17].

### 2.3. Quantification of MVs

MVs were quantified as lipid concentrations after staining with *N*-(3-Triethylammoniumpropyl)-4-(6-(4-(diethylamino) phenyl) hexatrienyl) pyridinium (FM4-64; Biotium, Fremont, CA, USA), as previously described [13]. Briefly, the samples were stained with 5 µM FM4-64, and the fluorescence intensity was measured using an SH-9000Lab microplate reader (excitation wavelength: 515 nm, fluorescence wavelength: 640 nm; Corona Electric, Ibaraki, Japan). Protein, DNA, and small RNA concentrations were determined using Qubit^TM^ protein assay kit (Thermo Fisher Scientific, Waltham, MA, USA), Qubit^TM^ DNA HS assay kit (Thermo Fisher Scientific), and Qubit^TM^ miRNA assay kit (Thermo Fisher Scientific), respectively. Small RNAs in the MVs were extracted using the miRNeasy Mini Kit (<200 nucleotides; Qiagen, Tokyo, Japan) according to the manufacturer’s instructions. 

### 2.4. Nanoparticle Tracking Analysis (NTA)

The diameter and concentration of the MVs were analyzed using a NanoSight NS300 system (Malvern Instruments, Malvern, UK). The MV suspension in HEPES-NaCl was loaded and analyzed using the NTA software Version 3.4.4 (Malvern Instruments). Each sample was recorded five times for 60 s at a constant temperature of 23 °C.

### 2.5. Cell Culture and Anti-Inflammatory Assay

HT29 cells (human adenocarcinoma cell line) were purchased from the European Collection of Authenticated Cell Cultures (ECACC, Salisbury, UK). Cells were maintained in Dulbecco’s modified Eagle’s medium (Fujifilm Wako Chemicals, Tokyo, Japan) containing 10% (*v*/*v*) fetal bovine serum (FBS; BioWest, Nuaillé, France), 100 units/mL penicillin, and 100 μg/mL streptomycin at 37 °C in the presence of 5% CO_2_.

The cells were inoculated in 48-well plates (Thermo Scientific) at a density of 2.5 × 10^4^ cells/well and were monolayered for 7-day cultivation, changing the medium every 3 days. The cells were preincubated with the samples for 30 min, and LPS (derived from *E. coli* O55:B5; Merck, Darmstadt, Germany) was subsequently added to each well at a final concentration of 1 ng/mL and cultured for 24 h. As a positive control for an anti-inflammatory effect, 20 nM BAY11-7085 (Tokyo Chemical Industry, Tokyo, Japan), which inhibits NF-*κ*B activation, was used.

The IL-8 concentration was determined using a human IL-8 Duoset^®^ ELISA development kit (R&D Systems, Minneapolis, MN, USA) and TMB microwell peroxidase substrate system (SeraCare, Milford, MA, USA), according to the manufacturer’s protocols. After stopping the reaction by adding 1 M sulfuric acid, the absorbance was measured at 450 nm using an infinite F200 microplate reader (Tecan, Männedorf, Switzerland).

### 2.6. HT29 Cell Viability Assay

HT29 cells were inoculated in 96-well plates at 5.0 × 10^4^ cells/well in the presence or absence of a sample and incubated for 24 h. Subsequently, 10 μL of WST-1 (Takara, Shiga, Japan) was added to each well and incubated at 37 °C in the presence of 5% CO_2_ for 30 min. The absorbance was measured at 450 nm using an infinite F200 microplate reader. The ratio of the absorbance of each tested group to that of the negative control group was calculated as cell viability.

### 2.7. Uptake of MVs by HT29 Cells

MVs were fluorescently labeled with 10 µM FM4-64 for 10 min at 37 °C and then washed with HEPES-NaCl using a Vivaspin 500 (MWCO 10,000; Sartorius, Göttingen, Germany). Bacterial cells were stained with 50 µM FM4-64 at 37 °C for 30 min and then washed with HEPES-NaCl via centrifugation (12,000× *g*, 5 min, 4 °C).

HT29 cells were monolayered on a culture cover glass. FM4-64-labeled MVs (50 µg-protein/well) or bacterial cells (50 µg-dry cell/well) were added to HT29 cells and then incubated. After washing with D-PBS (-), the cell membranes of HT29 cells was stained with 10 µg/mL fluorescein-4-isothiocyanate (FITC)-labeled wheat germ agglutinin (FITC-WGA; Biotium) at 37 °C for 10 min. After washing, the cells were incubated with pre-cooled (−20 °C) methanol for 10 min. After washing, the cells were incubated with 300 ng/mL Cellstain^®^DAPI (Dojindo Laboratories, Kumamoto, Japan) at 37 °C for 10 min. The cells were then embedded in Vectashield (Vector laboratories, Newark, CA, USA), and the signals of 4′,6-diamidino-2-phenylindole (DAPI) (dichroic mirror: 405 nm, bandpass emission filter: 429–474 nm), FITC (dichroic mirror: 488 nm, bandpass emission filter: 504–544 nm), and FM4-64 (dichroic mirror: 561 nm, bandpass emission filter: 649–750 nm) were subsequently observed using a confocal laser scanning microscope AX-R (Nikon, Tokyo, Japan).

### 2.8. Animal Experiments Using a DSS-Induced Colitis Mouse Model

Animal experiments were approved by the Kansai University Animal Experiment Committee (Approval No. 2221) and conducted in accordance with the Kansai University Animal Experiment Guidelines and the ARRIVE guidelines (https://arriveguidelines.org/, accessed on 5 April 2021). Male BALB/c mice were purchased from Japan SLC (Shizuoka, Japan) and reared as described previously [14]. Mice were pre-reared for one week and then used in the experiment at the age of 6 weeks. Two independent animal experiments were performed, and the results were merged for analysis. Mice were divided into three groups: control (number n = 5 + 6), DSS (n = 5 + 6), and DSS + *Lp*MV (n = 5 + 8). From the beginning of the experiment (day 0), the control group was fed water, while the DSS and DSS + *Lp*MV groups were fed 2.5% DSS solution (colitis grade, MW 36,000–50,000; MP Biomedicals, Irvine, CA, USA) ad libitum for 14 days. Starting on day 7, the DSS group was treated orally with PBS, whereas the DSS + *Lp*MV group was treated with *Lp*MVs (40 µg-protein/mouse) every day for 1 week. Body weight was monitored daily, and fecal conditions were monitored starting from day 7. The Disease Activity Index (DAI) was scored for each of the three parameters: rate of weight loss, shape of feces, and bleeding in feces, and the sum of the scores for the three parameters was calculated [18,19]. Occult blood in the feces was detected using the hemoglobin pseudoperoxidase activity method with Uliace Kc (Terumo, Tokyo, Japan). On day 14, all mice were euthanized by drawing blood from the abdominal aorta under isoflurane anesthesia.

### 2.9. Histopathological Analysis

Mice colon specimens were collected after sacrifice, fixed with 4% paraformaldehyde (pH 7.4) for 24 h at 25 °C, dehydrated, dealcoholized, and permeated with paraffin. They were then embedded in paraffin blocks and sectioned into 5 µm thick sections using a rotary tissue processor (TP1020; Leica Biosystems, GmbH, Wetzlar, Germany). The sections were stained with hematoxylin-eosin (HE) stain (Fujifilm Wako Chemicals) [20] and observed under a fluorescence microscope, BZ-X800 (Keyence, Osaka, Japan). Histopathological grading scores were used to evaluate IBD based on microscopic images of the colon sections [21].

### 2.10. Flow Cytometry Analysis of Lamina Propria Lymphocytes (LPL)

Briefly, diced colon tissues were incubated in RPMI-1640 containing 0.5 mM EDTA and 2% (*v*/*v*) FBS for 15 min at 37 °C and subsequently incubated in RPMI-1640 containing 2 mg/mL collagenase (Fujifilm Wako Chemicals) and 2% (*v*/*v*) FBS for 15 min at 37 °C. The tissues were crushed, and the cell suspension was centrifuged (390× *g*, 10 min, 4 °C). The precipitate was suspended in 40% (*v*/*v*) Percoll (Cytiva) in RPMI-1640 containing 2% (*v*/*v*) FBS, layered on a 75% (*v*/*v*) Percoll, and then centrifuged (810× *g*, 20 min, 20 °C). LPL cells that accumulated at the borders were collected.

LPL cells were stained with 5 µg/mL TruStain FcXTM (clone: 93; Biolegend, San Diego, CA, USA) and 0.5 µg/mL 7-amino-actinomycin D (7AAD) for 15 min at room temperature (18–23 °C). The cells were stained with 5 µg/mL FITC anti-mouse Ly6G (clone: 1A8; Biolegend), 2 µg/mL APC-Cy7 anti-mouse CD11b (clone: M1/70; Biolegend), and 2 µg/mL BV421 anti-mouse CD45 (clone: 30-F11; Biolegend) for 30 min, and then washed (400× *g*, 5 min, 4 °C) with PBS containing 2% (*v*/*v*) FBS. The percentage of CD11b^+^ Ly6G^+^ neutrophils among the total live cells (gated with 7AAD^−^ CD45^+^) was analyzed using MACSQuant (Miltenyi Biotec, Gladbach, Germany) and FlowJo (ver. 10; BD Biosciences, Franklin Lakes, NJ, USA).

### 2.11. Statistical Analysis

In vitro experimental data are expressed as the mean ± standard error and analyzed using Dunnett’s multiple comparison test in SPSS Statistics (version 27; IBM, Chicago, IL, USA). For the in vivo experimental data, Dunn’s nonparametric multiple comparison test or Holm–Sidak’s parametric multiple comparison test was performed using GraphPad Prism 6 (MDF, Tokyo, Japan). A *p* value of <0.05 was considered statistically significant.

## 3. Results

### 3.1. MV Production by Probiotics and the Corresponding Anti-Inflammatory Effects

**Figure 1:**
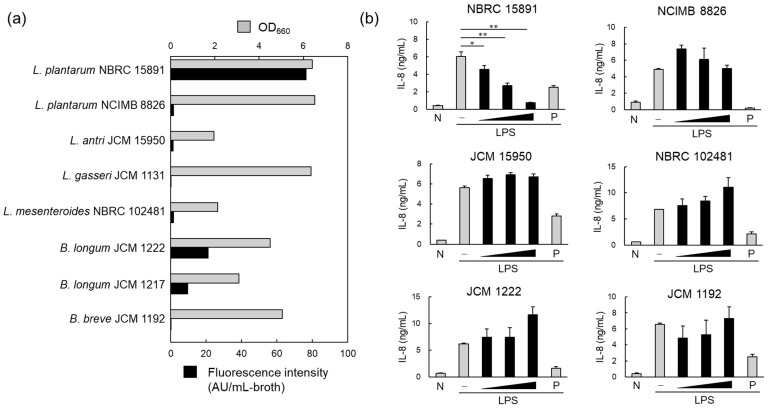
Membrane vesicle (MV) production and anti-inflammatory effects of MVs derived from probiotics. (**a**) MV production in lactic acid bacteria and bifidobacteria. Bacterial cells were cultured for 96 h, and the MVs collected from the culture supernatant using ultracentrifugation were quantified by measuring the fluorescence intensity of FM4-64. (**b**) Anti-inflammatory effects of MVs derived from probiotics were evaluated via the inhibition of IL-8 production in HT29 cells induced by inflammation with lipopolysaccharide (1 ng/mL). The black vertical bar indicates that MVs from the same volume of medium were added to HT29 cells in a 10-fold dilution series. Mean ± standard error (SE), n = 3. * and ** denote *p* < 0.05 and *p* < 0.01, respectively, using Dunnett’s test. N; negative control, P; positive control (20 nM BAY 11-7085).

To investigate the MV productivity of five strains of lactic acid bacteria and three strains of bifidobacteria, crude MVs (200 μL) were prepared from the culture supernatant (50 mL) after 72 h, and the lipid concentration was determined. The results showed that MV productivity varied among the tested strains, regardless of bacterial concentration. *L. plantarum* NBRC 15891 had the highest MV productivity, followed by *B. longum* JCM 1222 (Figure 1a). Additionally, there were differences in MV productivity even within the same species, as observed between *L. plantarum* NBRC 15891 and NCIMB 8826 (Figure 1a).

The anti-inflammatory effects of crude MVs derived from the six MV-producing strains were also investigated. IL-8 is a key proinflammatory cytokine that induces neutrophil trafficking. Therefore, the suppression of IL-8 production induced by LPS in HT29 cells, a human adenocarcinoma cell line, was used to evaluate the anti-inflammatory effects. When 200 μL of crude MV fractions, prepared from 50 mL of culture supernatant, was added to the cell culture in a 10-fold dilution series at 1/100 volume, only *Lp*MV showed a concentration-dependent suppression of IL-8 production (Figure 1b). 

### 3.2. Characteristics of MV Production of L. plantarum NBRC 15891

**Figure 2:**
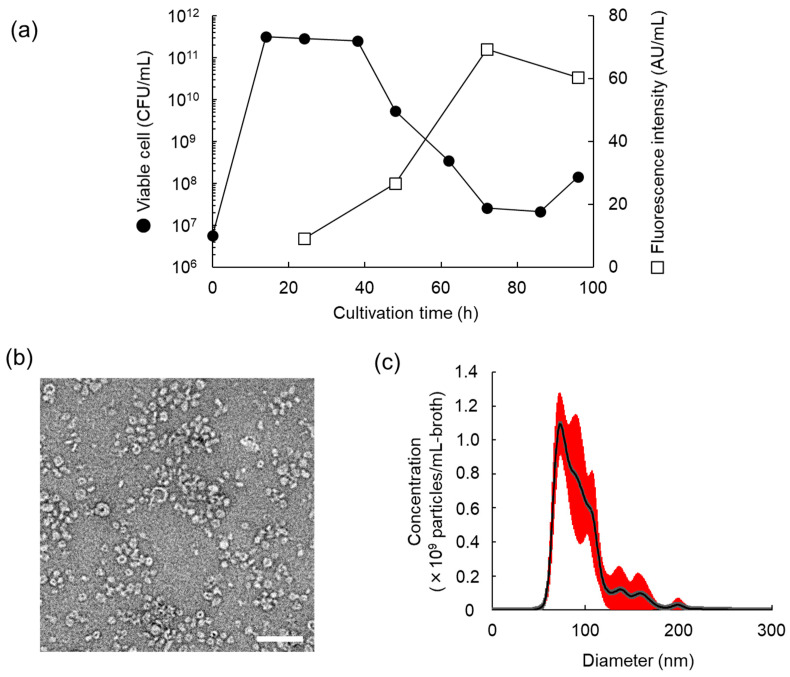
Properties of MV production of *Lactiplantibacillus plantarum* NBRC 15891. (**a**) Time course of MV production of *L. plantarum* NBRC 15891. MV concentration was measured using the fluorescence intensity of FM4-64. (**b**) Transmission electron microscope image of MVs derived from *L. plantarum* NBRC 15891 (*Lp*MVs) at 72 h. The scale bar indicates 200 nm. (**c**) Nanoparticle tracking analysis of *Lp*MVs at 72 h. Means ± SE (red error bars), n = 5.

To investigate the properties of MV production of *L. plantarum* NBRC 15891, a 200 µL crude MV suspension was prepared from 200 mL of culture supernatant at 24 h (early stationary phase), 48 h (early death phase), and 72 and 96 h (mid and late death phase), respectively. Results showed that MV concentration increases with cultivation time, with the highest concentration produced at 72 h, when viable cell concentrations rapidly decreased (Figure 2a). TEM observations revealed that purified *Lp*MVs obtained through gel filtration chromatography at 72 h of culture were heterogeneous spherical particles with a diameter of approximately 40–100 nm (Figure 2b). *Lp*MVs with diameters of approximately 70 nm were particularly abundant. The average particle diameter determined using NTA analysis was 94.6 ± 0.7 nm, and the particle concentration was 4.8 × 10^10^ particles/mL-broth (Figure 2c). The particle size peak was broad, suggesting that particles tended to aggregate with each other. Protein, small RNA, and DNA concentrations in 10^10^ particles of *Lp*MV were 16.7, 4.1, and 0.6 µg, respectively. 

### 3.3. Anti-Inflammatory Effects of LpMVs

**Figure 3:**
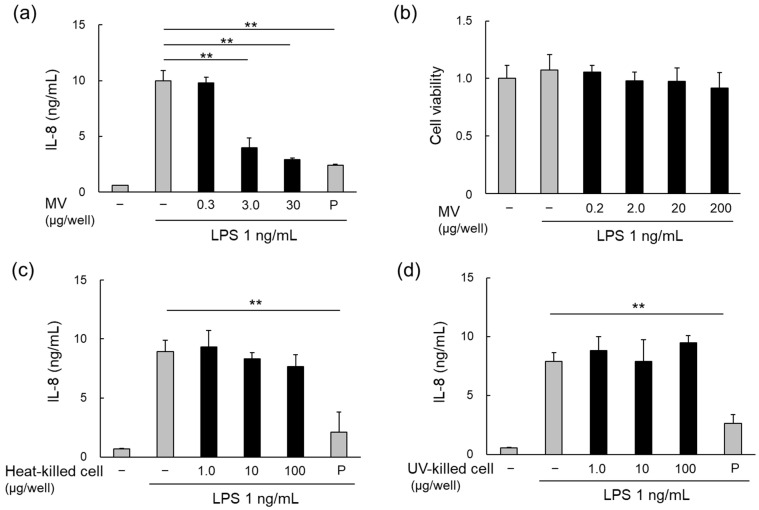
Anti-inflammatory effects of MVs and cells of *L. plantarum* NBRC 15891. (**a**) Anti-inflammatory effects of MVs derived from *L. plantarum* NBRC 15891 (*Lp*MVs), evaluated using HT29 cells. (**b**) Cytotoxicity test of *Lp*MVs using HT29 cells. Evaluation of anti-inflammatory effects of (**c**) heat- and (**d**) UV-killed cells of *L. plantarum* NBRC 15891 using HT29 cells. Data are expressed as mean ± standard error (SE), n = 3. ** denotes *p* < 0.01 using Dunnett’s test. N; negative control, P; positive control (20 nM BAY 11-7085).

Purified *Lp*MVs suppressed LPS-induced IL-8 production in HT29 cells in a concentration-dependent manner (Figure 3a) that was not due to cytotoxicity (Figure 3b). However, neither heat- nor UV-killed cells inhibited IL-8 production (Figure 3c,d). These results suggest that the anti-inflammatory effects are specific to MVs and are exerted by their release from their parent cells.

### 3.4. MV Uptake by HT29 Cells

**Figure 4:**
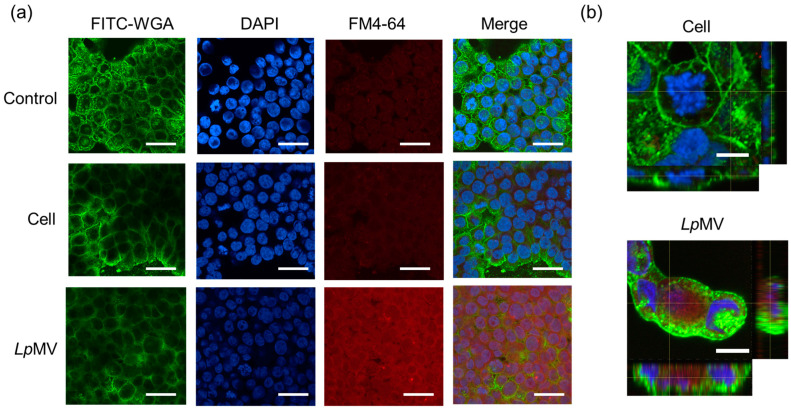
MV uptake ability of HT29 cells. (**a**) FM4-64-labeled *Lp*MVs (10 µg-protein/mL) or *L. plantarum* NBRC 15891 cells (10 µg-dry cells/mL) were added to HT29 cells and incubated for 20 h. Nuclei were stained with 4′,6-diamidino-2-phenylindole (DAPI), and cell membranes were stained with fluorescein-4-isothiocyanate (FITC)-labeled wheat germ agglutinin (WGA). The scale bars indicate 100 µm. (**b**) High magnification of HT29 cells incubated with cells and *Lp*MVs in X-Y, X-Z, and Y-Z directions. The scale bar represents 10 µm.

When HT29 cells were incubated with bacterial cells, no red signals from the cells were detected under a confocal laser microscope (Figure 4a). However, in HT29 cells incubated with *Lp*MVs, red signals were observed throughout the area where the cells were present (Figure 4a). The signals were weak when the incubation time with *Lp*MVs was 30 min and became stronger as the incubation time increased (Figure S1). Furthermore, observation in the depth direction revealed red signals inside the HT29 cell membrane (Figure 4b), indicating that *Lp*MVs were taken up by the cells, whereas bacterial cells were not. 

### 3.5. Inflammatory Effects of RNAs in LpMVs

**Figure 5:**
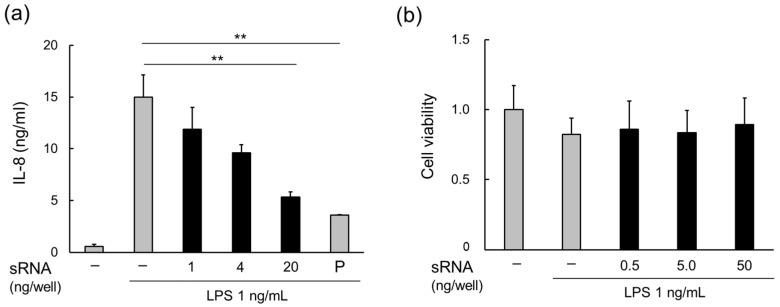
Anti-inflammatory effects of small RNAs in MVs derived from *L. plantarum* NBRC 15891 (*Lp*MVs). (**a**) Anti-inflammatory effects of small RNAs in *Lp*MVs evaluated using HT29 cells. (**b**) Cytotoxicity test of small RNAs in *Lp*MVs using HT29 cells. sRNA; small RNA, P; positive control (20 nM BAY 11-7085). Data are expressed as mean ± standard error (SE), n = 4. ** denotes *p* < 0.01 using Dunnett’s test.

MVs derived from *L. plantarum* JCM 8341 contain RNAs; however, the majority are small RNAs, mainly consisting of 5S, 16S, and 23S rRNA, as well as tRNA (smaller than 100 nt) [16]. As *Lp*MVs also contain RNA, small RNAs (<200 nt) were extracted from *Lp*MVs. The small RNAs suppressed LPS-induced inflammation on HT29 cells in a concentration-dependent manner (Figure 5a) and the RNAs did not exhibit cytotoxicity (Figure 5b). 

### 3.6. Anti-Inflammatory Effects of LpMVs in Colitis Mice

**Figure 6:**
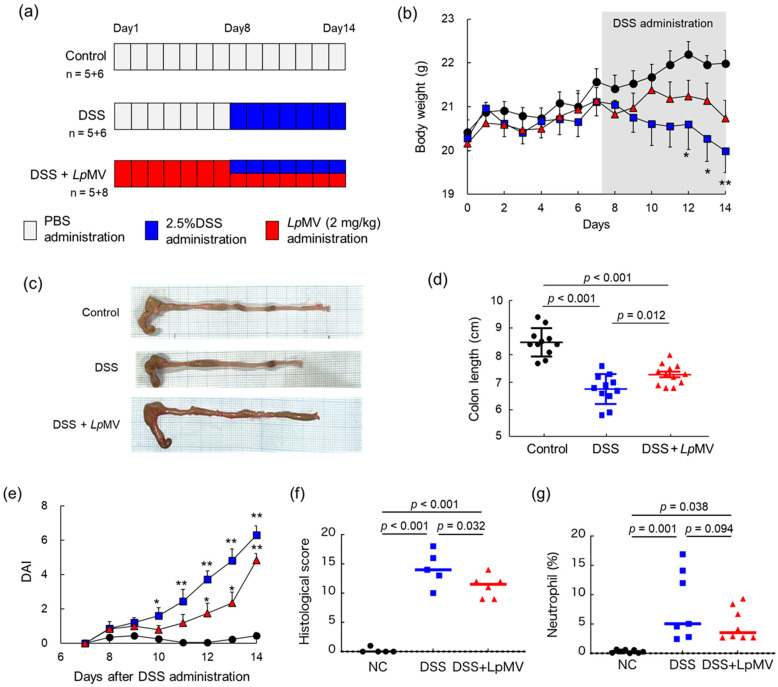
Evaluation of the anti-inflammatory effects of *Lp*MV using a dextran sulfate sodium (DSS)-induced colitis mouse model. (**a**) Schematic timeline of the animal experiment. (**b**) Changes in body weight of mice. Circles represent the control group (n = 11), squares represent the DSS group (n = 11), and triangles represent the DSS + MV group (n = 12). Data are expressed as mean ± SE. * and ** denote *p* < 0.05 and *p* < 0.01, respectively, vs. the control group after one-way ANOVA followed by Holm–Sidak’s multiple comparison test. (**c**) Typical colon of each experimental group. (**d**) Effects of *Lp*MV administration on colon length. The *p* values were calculated using one-way ANOVA followed by Holm–Sidak’s multiple comparison test. (**e**) Changes in DAI after DSS administration to mice. DAI was calculated for 7 days after DSS administration. Circles represent the control group (n = 11), squares represent the DSS group (n = 11), and triangles represent the DSS + MV group (n = 12). Data are expressed as mean ± SE. * and ** denote *p* < 0.05 and *p* < 0.01, respectively, for the control group as per the Kruskal–Wallis test followed by Dunn’s multiple comparison test. (**f**) Histological scores were calculated based on the histological evaluation scoring criteria. The *p* values were calculated using one-way ANOVA followed by Holm–Sidak’s multiple comparison test. (**g**) Percentage of CD11b^+^ Ly6G^+^ neutrophils in lamina propria lymphocytes. The *p* values were calculated using one-way ANOVA followed by Holm–Sidak’s multiple comparison test.

Two independent animal experiments were conducted (Figure 6a), and one mouse in the DSS + *Lp*MV group died from an unknown cause on day 9 of the first run. Prior to the administration of DSS, there was no significant difference in body weight among the groups, and administration of *Lp*MVs had no effect on body weight (Figure 6b). After day 8, the control group showed no weight loss. However, the DSS group gradually lost weight, which became more notable after day 12 (Figure 6b). In contrast, the DSS + *Lp*MV group showed no significant difference from the control group, and weight loss was suppressed compared to that in the DSS group (Figure 6b). Colon length was significantly shortened in the DSS (*p* < 0.001) and DSS + *Lp*MV groups (*p* < 0.001) compared to that in the control group, whereas colon length shortening was suppressed in the DSS + *Lp*MV group compared to that in the DSS group (*p* = 0.012) (Figure 6c,d). DAI was assessed based on body weight loss, stool consistency, and the presence of blood in the stool. The DAI increased significantly on day 10 in the DSS group compared to that in the control group (*p* < 0.05) and on all days after day 11 (*p* < 0.01) (Figure 6e and Figure S2a–c). In contrast, the DAI in the DSS + *Lp*MV group increased significantly on days 12 and 13 (*p* < 0.05) and on day 14 (*p* < 0.01) compared to that in the control group (Figure 6e). These results indicate that *Lp*MVs administration delayed the clinical symptoms of colitis. The histological grading score of the DSS and DSS + *Lp*MV groups was significantly higher than that of the control group (*p* < 0.001) and significantly lower in the DSS + *Lp*MV group than in the DSS group (*p* = 0.032) (Figure 6f and Figure S3). This suggests that administration of *Lp*MVs tends to improve histological symptoms. As *Lp*MVs suppressed IL-8 production in HT29 cells, we investigated whether *Lp*MVs also suppressed neutrophil accumulation in DSS-induced colitis mice. The percentage of neutrophils, defined as CD11b^+^ Ly-6G^+^ cells among LPL cells, was significantly increased in the DSS and DSS + *Lp*MV groups compared to that in the control group, while it tended to decrease in the DSS + *Lp*MV group compared to that in the DSS group (*p* = 0.094) (Figure 6g).

## 4. Discussion

Recently, the bioactivity of MVs released into host cells by commensal bacteria and probiotics has attracted considerable attention. In the present study, *L. plantarum* NBRC 15891 was selected as the probiotic that produces a large number of MVs with anti-inflammatory effects, and the characteristics of *Lp*MVs and some of the mechanisms by which *Lp*MVs exert anti-inflammatory effects were elucidated.

*L. plantarum* NBRC 15891 produced the highest amount of MVs among the four lactic acid bacteria and three bifidobacterial strains examined (Figure 1). This strain showed increased MV production in the early death phase, and this trend was also observed in *L. antri* JCM 15950^T^ (Figure 2a), suggesting that lysis by cell death is one of the triggers for the increased MV production in Gram-positive bacteria due to the extended culture time. The MVs of Gram-positive bacteria are formed when the plasma membrane is extruded through small pores in the cell wall. These pores are formed by cell-wall-degrading enzymes such as lysozyme and phage-derived endolysin [22]. Additionally, we reported that MV production in *L. antri* is promoted by glycine-induced cell wall weakening [15]. According to genome analysis, *L. plantarum* NBRC 15891 possesses 15 genes encoding lysozyme family proteins (GenBank accession number: NZ_ACGZ00000000); however, *L. gasseri* JCM 1131, which rarely produces MVs, also possesses 7 genes encoding them (GenBank accession number: NZ_JAQZAQ000000000). These findings suggest that the ability to produce MVs depends on differences in the expression levels or activities of cell-wall-degrading enzymes and the cell wall structures.

Furthermore, we demonstrated that *Lp*MVs, but not bacterial cells, have anti-inflammatory effects when LPS-induced IL-8 production in HT29 cells (Figure 3). Several *Lactobacillus* strains possess anti-inflammatory activity [23], and lipoteichoic acid [24,25] and extracellular polysaccharides [26,27] have been reported as active anti-inflammatory components of *Lactobacillus* strains prior to their reclassification in 2020 [28]. The anti-inflammatory effect of *Lp*MVs reported here is unique because it is specific to the MVs, and its mechanism is different from that of bacterial cells. Furthermore, the culture supernatants of several lactic acid bacteria have been reported to inhibit the production of inflammatory cytokines induced by LPS in HT29 cells [29]. Although the components and mechanisms of inhibition are unclear because the previous study did not focus on MVs, our study points out that it is necessary to focus not only on metabolites but also on MVs when considering the immunomodulatory effects of lactic acid bacteria.

The key differences between bacterial cells and MVs are their sizes and surface structures. Intestinal epithelial cells, including HT29 cells, can take up nanoparticles [30], and we were able to demonstrate that bacterial cells were not taken up by HT29 cells, whereas *Lp*MVs were (Figure 4). In addition, while the bacterial cells did not inhibit IL-8 production by HT29 cells, *Lp*MVs and the small RNAs extracted from *Lp*MVs inhibited IL-8 production (Figure 5). These results suggest that small RNAs act on HT29 cells by being encapsulated and released as MVs from the parent cells. MVs often serve as carriers for delivering cargo to host cells. For example, outer membrane vesicles (OMVs) produced by *Shigella flexneri* are taken up by human intestinal epithelial cells, which release nonmembrane-permeable antibiotics encapsulated in OMVs into the cytoplasm [31]. OMVs derived from *Pseudomonas aeruginosa* are taken up by host cells, and the DNA included in the OMVs is transferred into the cells [32]. Recently, new technologies have been reported to introduce nucleic acids into MVs derived from *E. coli* and *L. reuteri* via electroporation and deliver them to host cells [33]. Although the direct relationship between MV uptake and the anti-inflammatory effects was unclear in the present study, it is suggested that specific small RNAs with anti-inflammatory effects in the cytoplasm are transferred to HT29 cells via MVs, resulting in the suppression of inflammation. 

On the other hand, we elucidated that small RNAs derived from *Lp*MVs suppressed the LPS-induced production of IL-8 in HT29 cells (Figure 5). LPS is recognized by the TLR4 and MD/2 complex and activates the transcription factor NF-κB via the myeloid differentiation primary response gene (MyD88) [34]. TLR4 signaling has also been identified to be important in the development of colorectal cancer in colitis [35], and the inhibition of TLR4 signaling may be useful in protecting against colitis and colitis-associated cancer. Some lactic acid bacterial strains have been reported to inhibit TLR4 signaling [36,37], and the soluble proteins of *Lacticaseibacillus rhamnosus* GG strain downregulate the TLR4/MyD88 axis [38]. Moreover, its MVs also possess anti-inflammatory effects by inhibiting TLR4 signaling [39]. Meanwhile, the bacterial cells of *L. gasseri* OLL280 and its RNA inhibit T cell proliferation via the MyD88-dependent signaling pathway [40]. Furthermore, MVs derived from *L. plantarum* JCM 8341 contain small RNAs that are less than 100 nt in length, such as rRNA and tRNA [14]. Several microRNAs (miRNAs) regulate the functions of host immune cells. For example, let-7i inhibits the expression of TLR4 [41]. It has been suggested that *Lp*MVs contain RNAs that regulate TLR4 signaling; however, the RNA sequences and the mechanism by which RNA acts on HT29 cells are still unclear. It has been reported that calcium ions in the medium enable RNAs to particulate and transfer to cells [42]. Further studies are needed to clarify the specific RNAs in *Lp*MVs that mediate this anti-inflammatory effect.

Further, we demonstrated that *Lp*MVs alleviated the symptoms of DSS-induced colitis in mice and in vitro using HT29 cells (Figure 6). In particular, *Lp*MVs suppressed IL-8 production by HT29 cells and suppressed weight loss and shortening of colon length in DSS-induced colitis mice, suggesting that *Lp*MVs may be effective in preventing or alleviating colitis symptoms. The administration of several lactic acid bacteria, such as *Lactobacillus acidophilus* [43], *L. plantarum* [44], and *Levilactobacillus brevis* [45], alleviates the symptoms of colitis in DSS-induced colitis mice. In the case of *L. brevis* administration, its anti-inflammatory effects have been reported to be induced by the remodeling of the intestinal microbiota [41]. In the present study, in vivo experiments using DSS-colitis mice showed that *Lp*MVs alleviated the symptoms of colitis, whereas the migration of neutrophils tended to be suppressed. It is possible that IL-8 not only acts on neutrophils, but also on other cells, including macrophages. This can lead to the suppression of inflammation in the gut. Previous studies have reported that the administration of MVs derived from the intestinal bacteria *Akkermansia muciniphila* can affect the composition of the intestinal microbiota [46]. In the present study, the administration of *Lp*MVs may also have an impact on the intestinal microbiota, but further analysis is required to draw a conclusion. If we can clarify the effects of *Lp*MVs on the intestinal microbiota, new functionalities of *Lp*MVs as postbiotics may also be discovered.

## 5. Conclusions

We found that MVs released from *L. plantarum* NBRC 15891 suppress inflammation in intestinal epithelial cells via the cargo of small RNAs. We also demonstrated that *Lp*MVs were partially effective at alleviating the symptoms of DSS-induced colitis in mice. How this MV-mediated anti-inflammatory effect was observed only in this strain should be elucidated by identifying the specific RNAs that contribute to the anti-inflammatory effect. Overall, our results provide a new perspective on postbiotic functional studies in fermented foods and on host–bacterial interactions.

## Data Availability

Data associated with this study will be shared upon reasonable request from the corresponding authors.

## Supplementary Materials

The following supporting information can be downloaded at: https://www.mdpi.com/article/10.3390/foods13060967/s1, Figure S1: Time course of MVs uptake by HT29 cells. Figure S2: Changes in clinical parameters related to the Disease Activity Index (DAI) after the administration of dextran sodium sulfate (DSS) to mice. Figure S3: Colon sections and their histological evaluation; Table S1: Bacterial strains used in this study.

## References

[1] Alatab S., Sepanlou S.G., Ikuta K., Vahedi H., Bisignano C., Safiri S., Sadeghi A., Nixon M.R., Abdoli A., Abolhassani H. (2020). The global, regional, and national burden of inflammatory bowel disease in 195 countries and territories, 1990–2017: A systematic analysis for the global burden of disease study 2017. Lancet Gastroenterol. Hepatol..

[2] Ramos G.P., Papadakis K.A. (2019). Mechanisms of disease: Inflammatory bowel diseases. Mayo Clin. Proc..

[3] Roda G., Dotti I., Cai Z., Wang S., Li J. (2021). Treatment of inflammatory bowel disease: A comprehensive review. Front. Med..

[4] Mazzucchelli L., Hauser C., Zgraggen K., Wagner H., Hess M., Laissue J.A., Mueller C. (1994). Grade of active inflammation. Am. J. Pathol..

[5] Yao S., Zhao Z., Wang W., Liu X. (2021). *Bifidobacterium Longum*: Protection against inflammatory bowel disease. J. Immunol. Res..

[6] Saez-Lara M.J., Gomez-Llorente C., Plaza-Diaz J., Gil A. (2015). The role of probiotic lactic acid bacteria and bifidobacteria in the prevention and treatment of inflammatory bowel disease and other related diseases: A systematic review of randomized human clinical trials. Biomed. Res. Int..

[7] Fan L., Qi Y., Qu S., Chen X., Li A., Hendi M., Xu C., Wang L., Hou T., Si J. (2021). *B. Adolescentis* ameliorates chronic colitis by regulating Treg/Th2 response and gut microbiota remodeling. Gut Microbes.

[8] Yan S., Yang B., Ross R.P., Stanton C., Zhang H., Zhao J., Chen W. (2020). *Bifidobacterium longum* subsp. *longum* YS108R fermented milk alleviates DSS induced colitis via anti-inflammation, mucosal barrier maintenance and gut microbiota modulation. J. Funct. Foods.

[9] Lu Q., Guo Y., Yang G., Cui L., Wu Z., Zeng X., Pan D., Cai Z. (2022). Structure and anti-inflammation potential of lipoteichoic acids isolated from *Lactobacillus* strains. Foods.

[10] Liu M., Zhang X., Hao Y., Ding J., Shen J., Xue Z., Qui W., Li Z., Song Y., Zhang T. (2019). Protective Effects of a Novel Probiotic strain, *Lactococcus lactis* ML2018, in colitis: In vivo and in vitro evidence. Food Funct..

[11] Toyofuku M., Schild S., Kaparakis-Liaskos M., Eberl L. (2023). Composition and functions of bacterial membrane vesicles. Nat. Rev. Microbiol..

[12] Toyofuku M., Tashiro Y., Hasegawa Y., Kurosawa M., Nomura N. (2015). Bacterial membrane vesicles, an overlooked environmental colloid: Biology, environmental perspectives and applications. Adv. Colloid. Interface Sci..

[13] Kurata A., Yamasaki-Yashiki S., Imai T., Miyazaki A., Watanabe K., Uegaki K. (2023). Enhancement of IgA production by membrane vesicles derived from *Bifidobacterium longum* subsp. infantis. Biosci. Biotechnol. Biochem..

[14] Yamasaki-Yashiki S., Miyoshi Y., Nakayama T., Kunisawa J., Katakura Y. (2019). IgA-enhancing effects of membrane vesicles derived from *Lactobacillus sakei* subsp. *sakei* NBRC15893. Biosci. Microbiota Food Health.

[15] Yamasaki-Yashiki S., Sakamoto Y., Nishimura K., Saika A., Ito T., Kunisawa J., Katakura Y. (2024). High productivity of immunostimulatory membrane vesicles of *Limosilactobacillus antri* using glycine. Biosci. Microbiota Food Health.

[16] Kurata A., Kiyohara S., Imai T., Yamasaki-Yashiki S., Zaima N., Moriyama T., Kishimoto N., Uegaki K. (2022). Characterization of extracellular vesicles from *Lactiplantibacillus plantarum*. Sci. Rep..

[17] Miyoshi Y., Saika A., Nagatake T., Matsunaga A., Kunisawa J., Katakura Y., Yamasaki-Yashiki S. (2021). Mechanisms underlying enhanced IgA production in Peyer’s patch cells by membrane vesicles derived from *Lactobacillus sakei*. Biosci. Biotechnol. Biochem..

[18] Bang B., Lichtenberger L.M. (2016). Methods of inducing inflammatory bowel disease in mice. Curr. Protoc. Pharmacol..

[19] Zhou F., Hamza T., Fleur A.S., Zhang Y., Yu H., Chen K., Heath J.E., Chen Y., Huang H., Feng H. (2018). Mice with inflammatory bowel disease are susceptible to Clostridium difficile infection with severe disease outcomes. Inflamm. Bowel Dis..

[20] Nagatake T., Shiogama Y., Inoue A., Kikuta J., Honda T., Tiwari P., Kishi T., Yanagisawa A., Isobe Y., Matsumoto N. (2018). The 17,18-Epoxyeicosatetraenoic acid–G protein–coupled receptor 40 axis ameliorates contact hypersensitivity by inhibiting neutrophil mobility in mice and cynomolgus macaques. J. Allergy Clin. Immunol..

[21] Koelink P.J., Wildenberg M.E., Stitt L.W., Feagan B.G., Koldijk M., van’t Wout A.B., Atreya R., Vieth M., Brandse J.F., Duijst S. (2018). Development of reliable, valid and responsive scoring systems for endoscopy and histology in animal models for inflammatory bowel disease. J. Crohns Colitis.

[22] Toyofuku M., Cárcamo-Oyarce G., Yamamoto T., Eisenstein F., Hsiao C.C., Kurosawa M., Gademann K., Pilhofer M., Nomura N., Eberl L. (2017). Prophage-triggered membrane vesicle formation through peptidoglycan damage in *Bacillus subtilis*. Nat. Commun..

[23] Piqué N., Berlanga M., Miñana-Galbis D. (2019). Health benefits of heat-killed (Tyndallized) probiotics: An overview. Int. J. Mol. Sci..

[24] Noh S.Y., Kang S.S., Yun C.H., Han S.H. (2015). Lipoteichoic acid from *Lactobacillus plantarum* inhibits Pam2CSK4-induced IL-8 production in human intestinal epithelial cells. Mol. Immunol..

[25] Kim K.W., Kang S.S., Woo S.J., Park O.J., Ahn K.B., Song K.D., Lee H.K., Yun C.H., Han S.H. (2017). Lipoteichoic acid of probiotic Lactobacillus plantarum attenuates Poly I:C-induced IL-8 production in porcine intestinal epithelial cells. Front. Microbiol..

[26] Kwon M., Lee J., Park S., Kwon O.H., Seo J., Roh S. (2020). Exopolysaccharide isolated from *Lactobacillus plantarum* L-14 has anti-inflammatory effects via the toll-like receptor 4 pathway in LPS-induced RAW 264.7 cells. Int. J. Mol. Sci..

[27] Taylan O., Yilmaz M.T., Dertli E. (2019). Partial Characterization of a levan type exopolysaccharide (EPS) produced by *Leuconostoc mesenteroides* showing immunostimulatory and antioxidant activities. Int. J. Biol. Macromol..

[28] Zheng J., Wittouck S., Salvetti E., Franz C.M.A.P., Harris H.M.B., Mattarelli P., O’toole P.W., Pot B., Vandamme P., Walter J. (2020). A taxonomic note on the genus *Lactobacillus*: Description of 23 novel genera, emended description of the genus *Lactobacillus beijerinck* 1901, and union of *Lactobacillaceae* and *Leuconostocaceae*. Int. J. Syst. Evol. Microbiol..

[29] De Marco S., Sichetti M., Muradyan D., Piccioni M., Traina G., Pagiotti R., Pietrella D. (2018). Probiotic cell-free supernatants exhibited anti-inflammatory and antioxidant activity on human gut epithelial cells and macrophages stimulated with LPS. J. Evid. Based Complement. Altern. Med..

[30] Yao M., He L., McClements D.J., Xiao H. (2015). Uptake of gold nanoparticles by intestinal epithelial cells: Impact of particle size on their absorption, accumulation, and toxicity. J. Agric. Food Chem..

[31] Kadurugamuwa J.L., Beveridge T.J. (1998). Delivery of the non-membrane-permeative antibiotic gentamicin into mammalian cells by using Shigella flexneri membrane vesicles. Antimicrob. Agents Chemother..

[32] Bitto N.J., Chapman R., Pidot S., Costin A., Lo C., Choi J., D’Cruze T., Reynolds E.C., Dashper S.G., Turnbull L. (2017). Bacterial membrane vesicles transport their DNA cargo into host cells. Sci. Rep..

[33] Kim S.I., Ha J.Y., Choi S.Y., Hong S.H., Lee H.J. (2022). Use of bacterial extracellular vesicles for gene delivery to host cells. Biomolecules.

[34] Ciesielska A., Matyjek M., Kwiatkowska K. (2021). TLR4 and CD14 Trafficking and its influence on LPS-induced pro-inflammatory signaling. Cell Mol. Life Sci..

[35] Fukata M., Chen A., Vamadevan A.S., Cohen J., Breglio K., Krishnareddy S., Hsu D., Xu R., Harpaz N., Dannenberg A.J. (2007). Toll-like receptor-4 promotes the development of colitis-associated colorectal tumors. Gastroenterology.

[36] Kanmani P., Kim H. (2018). Protective Effects of lactic acid bacteria against TLR4 induced inflammatory response in hepatoma HepG2 cells through modulation of toll-like receptor negative regulators of mitogen-activated protein kinase and NF-κB signaling. Front. Immunol..

[37] Shi J., Li H., Liang S., Evivie S.E., Huo G., Li B., Liu F. (2022). Selected Lactobacilli strains inhibit inflammation in LPS-induced RAW264.7 macrophages by suppressing the TLR4-mediated NF-κB and MAPKs activation. Food Sci. Technol..

[38] Li Y., Yang S., Lun J., Gao J., Gao X., Gong Z., Wan Y., He X., Cao H. (2020). Inhibitory effects of the *Lactobacillus rhamnosus* GG effector protein HM0539 on inflammatory response through the TLR4/MyD88/NF-KB axis. Front. Immunol..

[39] Tong L., Zhang X., Hao H., Liu Q., Zhou Z., Liang X., Liu T., Gong P., Zhang L., Zhai Z. (2021). *Lactobacillus rhamnosus* GG derived extracellular vesicles modulate gut microbiota and attenuate inflammatory in DSS-induced colitis mice. Nutrients.

[40] Yoshida A., Yamada K., Yamazaki Y., Sashihara T., Ikegami S., Shimizu M., Totsuka M. (2011). *Lactobacillus gasseri* OLL2809 and its RNA suppress proliferation of CD4+ T cells through a MyD88-dependent signalling pathway. Immunology.

[41] Bayraktar R., Bertilaccio M.T.S., Calin G.A. (2019). The interaction between two worlds: MicroRNAs and toll-like receptors. Front. Immunol..

[42] Hori S.I., Yamamoto T., Waki R., Wada S., Wada F., Noda M., Obika S. (2015). Ca^2+^ enrichment in culture medium potentiates effect of oligonucleotides. Nucleic Acids Res..

[43] Kim W.K., Han D.H., Jang Y.J., Park S., Jang S.J., Lee G., Han H.S., Ko G. (2021). Alleviation of DSS-induced colitis via *Lactobacillus acidophilus* treatment in mice. Food Funct..

[44] Sun M., Liu Y., Song Y., Gao Y., Zhao F., Luo Y., Qian F., Mu G., Tuo Y. (2020). The ameliorative effect of *Lactobacillus plantarum*-12 on DSS-induced murine colitis. Food Funct..

[45] Ding S., Ma Y., Liu G., Yan W., Jiang H., Fang J. (2019). *Lactobacillus brevis* alleviates DSS-induced colitis by reprograming intestinal microbiota and influencing serum metabolome in murine model. Front. Physiol..

[46] Wang X., Lin S., Wang L., Cao Z., Zhang M., Zhang Y., Liu R., Liu J. (2023). Versatility of bacterial outer membrane vesicles in regulating intestinal homeostasis. Sci. Ave..

