# Supplementary material for: RNA-Based Anti-Inflammatory Effects of Membrane Vesicles Derived from Lactiplantibacillus plantarum
Source: Foods. 2024 Mar 21;13(6):967. doi: 10.3390/foods13060967 (PMC10969829; doi:10.3390/foods13060967)
Supplement: Supplementary file 1 [file foods-13-00967-s001.zip › Figure S1-3_S.Yamasaki-Yashiki.pdf]

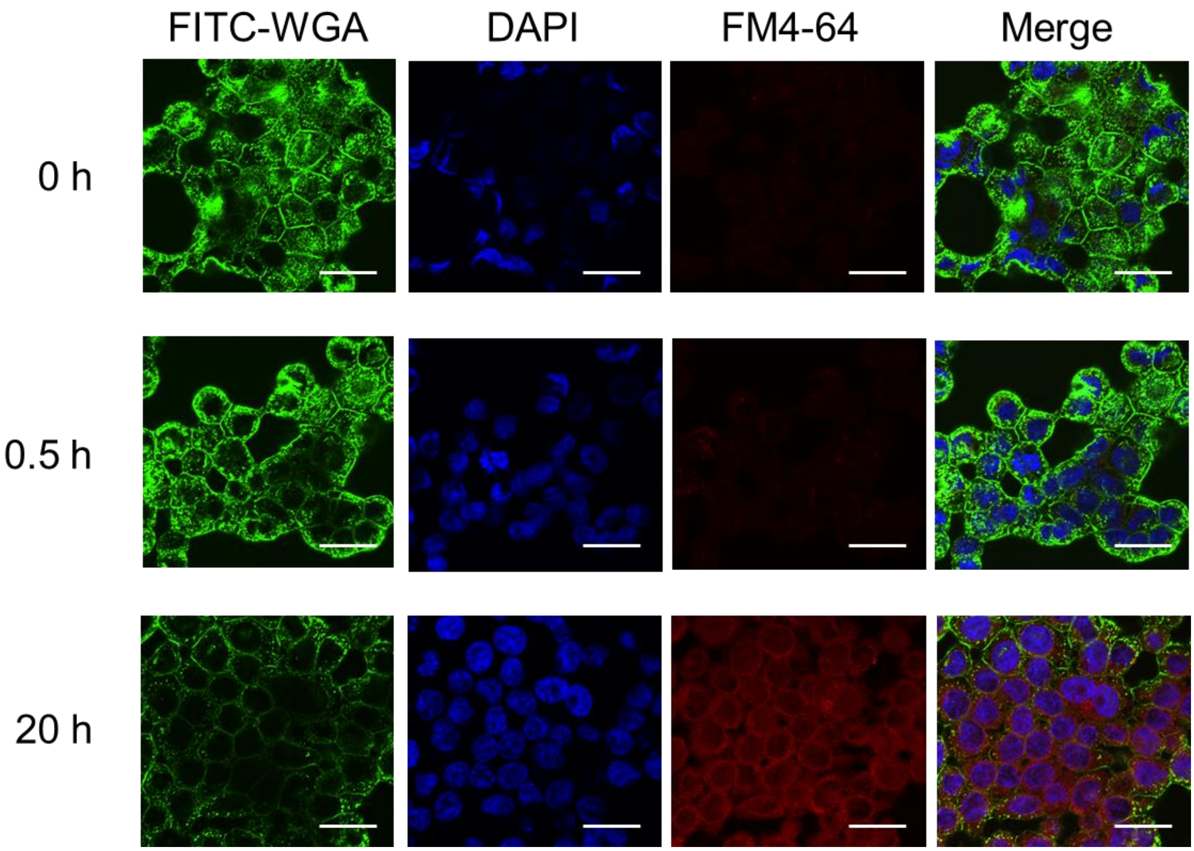

Figure S1. Time course of MV uptake by HT29 cells. FM4-64-labeled *Lp*MVs (10 μg-protein/mL) were added to HT29 cells and incubated for 0.5 and 20 h. Nuclei were stained with DAPI, and cell membranes were stained with FITC-labeled wheat germ agglutinin (WGA). Scale bars indicate 100 μm.

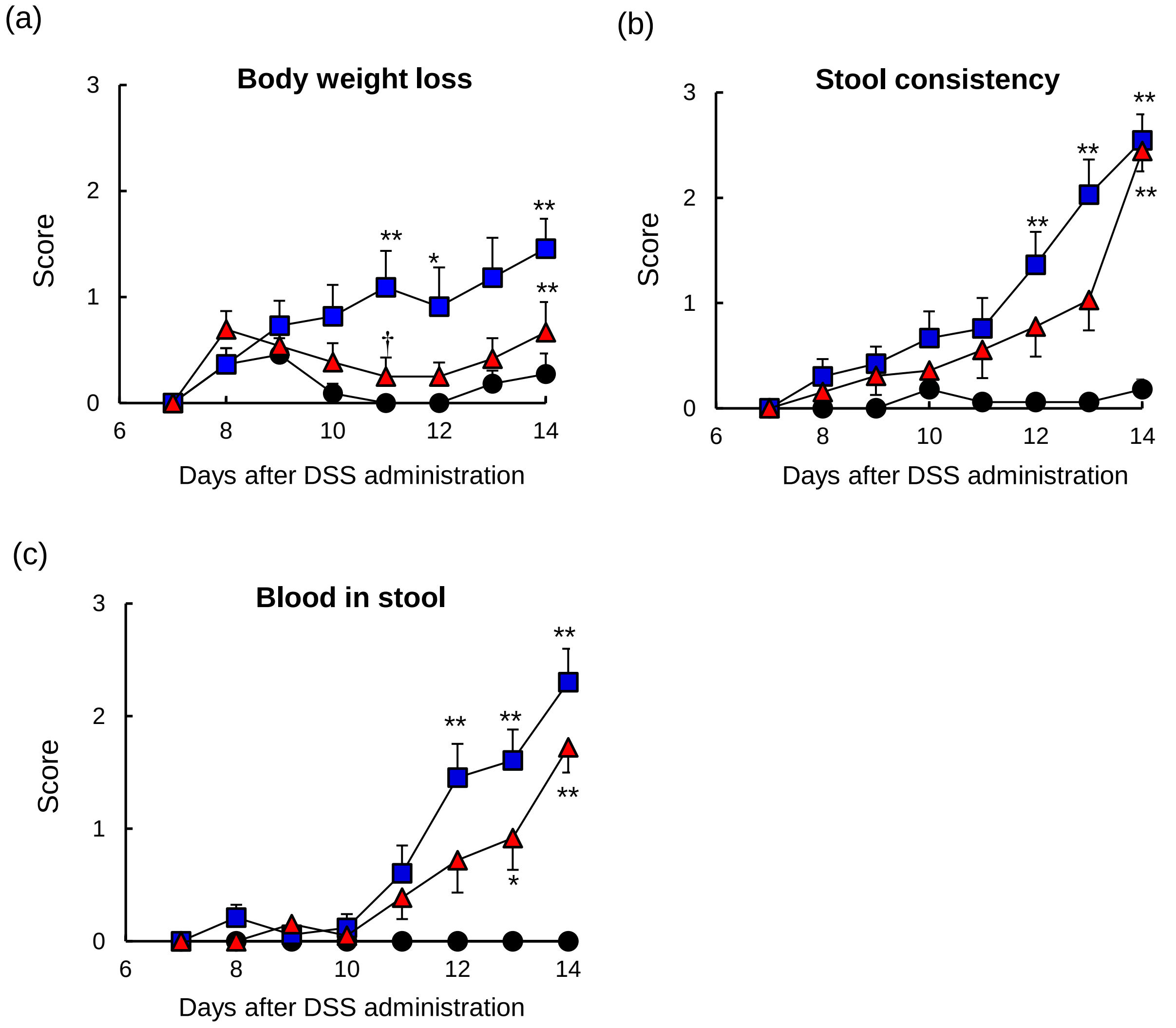

Figure S2. Changes in clinical parameters related to the disease activity index (DAI) after dextran sodium sulfate (DSS) administration to mice. Circles represent the control group (n = 11), squares represent the DSS group (n = 11), and triangles represent the DSS+MV group (n = 12). Data are expressed as mean ± SE. * and ** denote $p < 0.05$ and $p < 0.01$, respectively, vs. the control group as per Kruskal-Wallis test followed by Dunn's multiple comparison test.

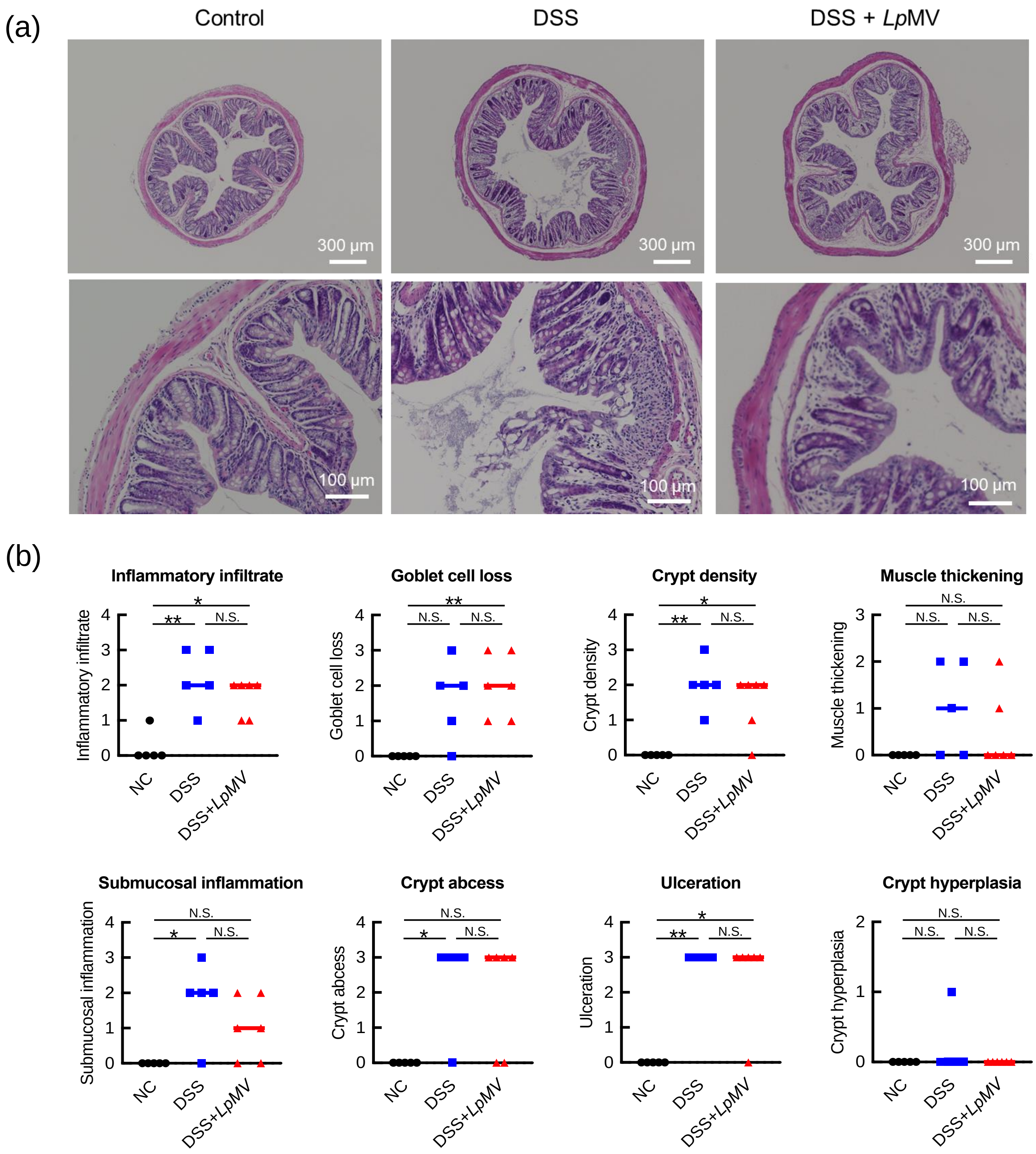

Figure S3. Colon sections and their histological evaluation. (a) Representative colon sections derived from mice on day 14 were stained with hematoxylin-eosin. (b) Histopathological grading scores were calculated based on the histological evaluation scoring criteria [18, 19]. * and ** denote $p < 0.05$ and $p < 0.01$, respectively, using Kruskal–Wallis test followed by Dunn's multiple comparison test. NC; Negative control.
