# Supplementary material for: RNA-Based Anti-Inflammatory Effects of Membrane Vesicles Derived from Lactiplantibacillus plantarum
Source: Foods. 2024 Mar 21;13(6):967. doi: 10.3390/foods13060967 (PMC10969829; doi:10.3390/foods13060967)
Supplement: Supplementary file 1 [file foods-13-00967-s001.zip › Table S1_S.Yamasaki-Yashiki.pdf]

Table S1. Bacterial strains used in this study

Strain	Source	Culture temperature (°C)
Lactiplantibacillus plantarum subsp. *plantarum* NBRC 15891^{T}	Pickled cabbage	30
Lactiplantibacillus plantarum NCIMB 8826	Human saliva	37
Limosilactobacillus antri JCM 15950^{T}	Human stomach mucosa	37
Lactobacillus gasseri JCM 1131^{T}	Human intestine	37
Leuconostoc mesenteroides subsp. *sake* NBRC 102481	Moto (seed mash for sake fermentation)	30
Bifidobacterium longum subsp. *infantis* JCM 1222^{T}	Intestine of infant	37
Bifidobacterium longum subsp. *longum* JCM 1217^{T}	Intestine of adult	37
Bifidobacterium breve JCM 1192^{T}	Intestine of infant	37
